# Complete genome sequence of *Brachyspira intermedia *reveals unique genomic features in *Brachyspira *species and phage-mediated horizontal gene transfer

**DOI:** 10.1186/1471-2164-12-395

**Published:** 2011-08-04

**Authors:** Therese Håfström, Désirée S Jansson, Bo Segerman

**Affiliations:** 1 Department of Bacteriology, National Veterinary Institute (SVA), SE 751 89 Uppsala, Sweden; 2 Department of Biomedical Sciences and Veterinary Public Health, Swedish University of Agricultural Sciences (SLU), SE 750 07 Uppsala, Sweden; 3 Department of Animal Health and Antimicrobial Strategies, National Veterinary Institute (SVA), SE 751 89 Uppsala, Sweden

**Keywords:** *Brachyspira intermedia*, genome comparison, bacteriophages, horizontal gene transfer, AT/GC-skew

## Abstract

**Background:**

*Brachyspira *spp. colonize the intestines of some mammalian and avian species and show different degrees of enteropathogenicity. *Brachyspira intermedia *can cause production losses in chickens and strain PWS/A^T ^now becomes the fourth genome to be completed in the genus *Brachyspira*.

**Results:**

15 classes of unique and shared genes were analyzed in *B. intermedia, B. murdochii, B. hyodysenteriae *and *B. pilosicoli*. The largest number of unique genes was found in *B. intermedia *and *B. murdochii*. This indicates the presence of larger pan-genomes. In general, hypothetical protein annotations are overrepresented among the unique genes. A 3.2 kb plasmid was found in *B. intermedia *strain PWS/A^T^. The plasmid was also present in the *B. murdochii *strain but not in nine other *Brachyspira *isolates. Within the *Brachyspira *genomes, genes had been translocated and also frequently switched between leading and lagging strands, a process that can be followed by different AT-skews in the third positions of synonymous codons. We also found evidence that bacteriophages were being remodeled and genes incorporated into them.

**Conclusions:**

The accessory gene pool shapes species-specific traits. It is also influenced by reductive genome evolution and horizontal gene transfer. Gene-transfer events can cross both species and genus boundaries and bacteriophages appear to play an important role in this process. A mechanism for horizontal gene transfer appears to be gene translocations leading to remodeling of bacteriophages in combination with broad tropism.

## Background

The genus *Brachyspira *currently comprises seven validly published species: *B. aalborgi*, *B. alvinipulli*, *B. hyodysenteriae*, *B. innocens*, *B. intermedia*, *B. murdochii *and *B. pilosicoli*. Collectively these form a distinct evolutionary line within the phylum *Spirochaetes *[1]. *Brachyspira *spp. are oxygen-tolerant anaerobes that colonize the intestines of some mammalian and avian species but they differ in enteropathogenicity from important pathogens of livestock to presumed commensals. *B. hyodysenteriae *and *B. pilosicoli *are important porcine pathogens, causing swine dysentery and porcine intestinal spirochetosis respectively. Two species, *B. pilosicoli *and *B. aalborgi*, can colonize humans and are suspected causes of colitis. For a review, see Tsinganou and Gebbers [2].

*B. intermedia *commonly colonize the large intestine of commercially farmed pigs and chickens and has also been isolated from rodents [3] and ducks (Jansson, D. unpublished data). The species was originally named *Serpulina intermedia *and described as possessing characteristics related to both the pathogen *B. hyodysenteriae *and the commensal *B. innocens *[4]. Later the species was transferred to the genus *Brachyspira *[5]. Data from field studies have suggested that *B. intermedia *may be a mild enteropathogen of pigs [6-8], but experimental challenge in porcine isolates has failed to produce clinical disease [9-11]. In contrast, an experimental challenge in chickens with *B. intermedia *isolates caused diarrhea, slow growth, and reduced egg production. [12-14]. Diagnostically relevant features associated with both *B. hyodysenteriae *and *B. intermedia *include tryptophanase and β-glucosidase activity. In addition, they lack α-galactosidase activity and are unable to hydrolyze hippurate [4,7]. The two species can be differentiated by haemolytic properties, *i.e.*, there is strong *β*-haemolysis in *B. hyodysenteriae *and weak *β*-haemolysis in *B. intermedia*. Other recognized representatives of the genus *Brachyspira *lack tryptophanase activity. Identification of isolates of *B. hyodysenteriae*, *B. intermedia *and the proposed species "*B. suanatina*" [15] by 16S rRNA gene- sequence analysis is not feasible because they form a common phylogenetic cluster [16]. Moreover, the high genetic diversity between isolates as seen from the results of pulsed-field gel electrophoresis, multi-locus sequence typing, and E-burst analysis have recently challenged the species delineation of weakly *β*-haemolytic and tryptophanase-producing isolates as *B. intermedia. *[17,18]. It remains to be seen whether all isolates with the phenotype described as characteristic for *B. intermedia *will be included in this species in the future.

Complete genome data from the two important porcine pathogens *B. hyodysenteriae *and *B. pilosicoli *and the presumed commensal *B. murdochii *have been recently published [19-21]. The *B. hyodysenteriae *genome consists of a 3 Mb chromosome and a ~36 kb plasmid. Many of these genes are more related to *Clostridium *and *Escherichia *species than to non-*Brachyspira *spirochetes which suggests that horizontal gene-transfer events have taken place [19]. The 36 kb plasmid is conserved among a large number of *B. hyodysenteriae *isolates but is not detected in any avirulent field strain, which suggests that it is important for virulence [22]. The only recognized gene-transfer agent in *Brachyspira *spp. is a mitomycin-inducible defective prophage (VSH-1), which transducts 7.5 kb random genomic fragments [23]. The *B. pilosicoli *genome is somewhat smaller, 2.6 Mb, and contains no plasmids. The *B. pilosicoli *genome has been compared to *B. hyodysenteriae *and a draft version of *B. murdochii *[21]. In our study the genome of *B. intermedia *type strain PWS/A^T ^(ATCC 51140) of porcine origin was completed and subjected to comparative genomic analysis. With this new data we can now compare four completed *Brachyspira *genomes and significantly increase the understanding of different and shared properties of the genomes. We also discuss horizontal movements of genes and the potential involvement of bacteriophages in this process.

## Results and discussion

### General genomic features and genome plasticity

**Figure 1:**
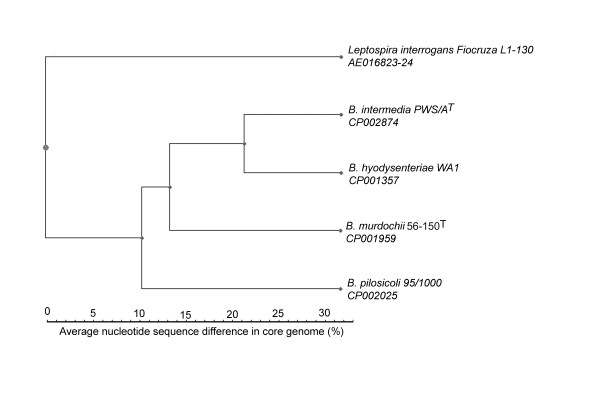
**Phylogenomic analysis**. Rooted Phylogenetic tree of *B. intermedia *PWS/A^T^, *B. hyodysenteriae *WA1, *B. murdochii *56-150^T^, *B. pilosicoli *95/1000 and *Leptospira interrogans *serovar Copenhageni str. Fiocruz L1-130, chromosome I and II. Accession numbers are shown in the figure. Distances were measured by the Average Similarity of the conserved Core genome (ASC) method and the tree was constructed using the UPGMA method.

The *B. intermedia *strain PWS/A^T ^genome consisted of a single circular 3,304,788 base pairs (bp) chromosome and a 3,260 bp plasmid (Table 1). The phylogenetic distances to the other available genomes within the *Brachyspira *genus and to the spirochete *Leptospira interrogans *(AE016823-24), calculated from average nucleotide similarity of the whole core genome [24], showed that *B. intermedia *strain PWS/A^T^ was most closely related to *B. hyodysenteriae *followed by *B. murdochii*; it was somewhat more distantly related to the *B. pilosicoli *genome (Figure 1). Also, average similarity distances indicated that the *B. pilosicoli *genome was closest to *B. murdochii *followed by *B. intermedia *and furthest from *B. hyodysenteriae*. This was in concordance with 16S rRNA gene-based phylogeny [21]. The size of the chromosome was only slightly larger than that of *B. murdochii *56-150^T^ (3,241,804 bp), somewhat larger than that of *B. hyodysenteriae *WA1 (3,000,694 bp) and noticeably larger than that of *B. pilosicoli *(2,586,443 bp). A total of 2,870 protein-coding genes (average length of 970 bp) were predicted in the chromosome. In addition 33 tRNA genes and single copies of the ribosomal RNA genes 5S (rrf), 16S (rrs) and 23S (rrl) were present. Comparisons to the other sequenced *Brachyspira *replicons are shown in Table 1.

**Table 1:** General genome features

Feature	***B. intermedia *PWS/A**^**T**^	***B. intermedia *PWS/A**^**T **^**Plasmid**	*B. hyodysenteriae *WA1	*B. hyodysenteriae *WA1 Plasmid	***B. murdochii *56-150**^**T**^	*B. pilosicoli *95/1000
Size (bp)	3,304,788	3,260	3,000,694	35,940	3,241,804	2,586,443
Coding region (%)	85.0	53.0	86.7	91.2	85.9	88.6
G+C content (%)	27.2	21.0	27.1	22.4	27.6	27.9
A+T content (%)	72.8	79.0	72.9	77.6	72.3	72.1
Average CDS length (bp)	970	578	996	1,058	992	997
Number of CDS	2,870	3	2,613	31	2,809	2,299
Assigned function	1,854	0	1,755	29	1,993	1,615
Conserved -/hypothetical	1,016	3	858	2	816	684
rRNA	3	0	3	0	3	3
tRNA	33	0	34	0	34	33

The G+C content for the *B. intermedia *strain PWS/A^T^ chromosome was 27.2% which is similar to that of the other genomes in the genus. Interestingly, the small plasmid had only 21.0% G+C content (Table 1). The G+C content in intergenic regions was 20.4%, and in the third positions of synonymous codons it was less than 10%. This indicates that Gs and Cs are shunned and the average G+C content is determined by the requirements of the G+C-containing codons. The small plasmid had a lower coding density and therefore also a lower G+C content.

**Figure 2:**
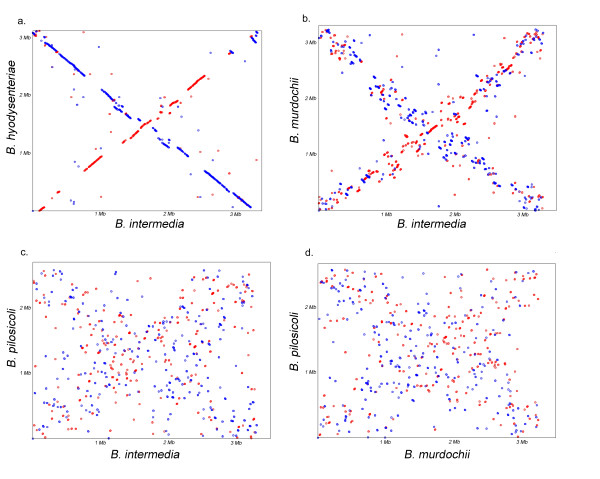
**Replication-directed translocations**. Whole genome alignments of *Brachyspira *species chromosomes. Sequences were permutated to set the origin of replication according to GC-skew. Red dots are conserved regions oriented in the same direction in both genomes and blue dots have an opposite orientation. a, *B. intermedia *PWS/A^T ^versus *B. hyodysenteriae *WA1. b, *B. intermedia *PWS/A^T ^versus *B. murdochii *56-150^T^. c, *B. intermedia *PWS/A^T ^versus *B. pilosicoli *95/1000. d, *B. murdochii *56-150^T ^versus *B. pilosicoli *95/1000.

**Figure 3:**
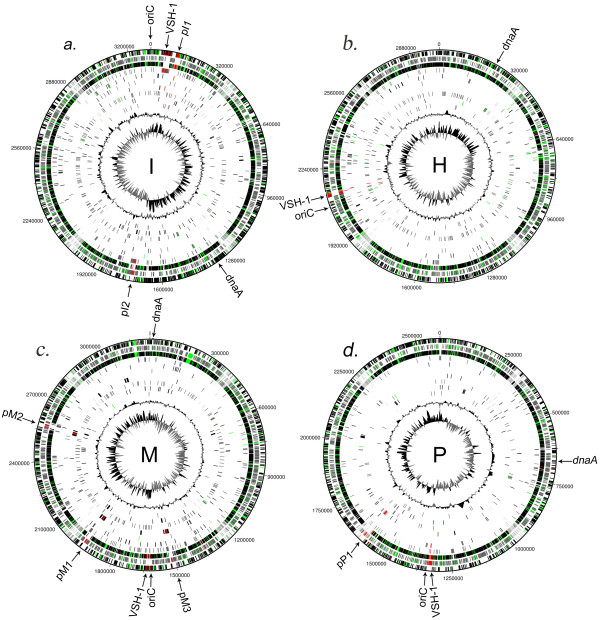
**Circular genome representations**. Circular representations of the available *Brachyspira *spp. genomes using DNAplotter. a, *B. intermedia *PWS/A^T^; b, *B. hyodysenteriae *WA1; c, *B. murdochii *56-150^T^; d, *B. pilosicoli *95/1000. The first two circles starting from the center in a-d are GC-skew and G+C content. The other eight circles starting after G+C content are circles showing genes conserved and shared in the four Brachyspira species: a, IHP, IMP, IHM, IP, IM, IH, I, IHMP; b, IHP, HMP, IHM, HP, HM, IH, H, IHMP; c, HMP, IMP, HIM, MP, MH, MI, M, IHMP; d, HMP, IMP, IHP, MP, HP,IP,P IHMP. The second circle from the outside shows all reverse strand genes. The outermost circle shows all forward strand genes in all four figures. The red- and green-colored genes are putative phage genes and putative virulence genes respectively.

The approximate origin of replication on the chromosome was located on the basis of the GC-skew pattern and the sequence file was permuted to start at this point. The leading strands (LES), defined as the plus strand from the first half of the genome and the minus strand from the second half, were enriched in Gs and also weakly enriched in Ts (Table 2). The origin of replication has previously been assumed to be adjacent to the *dnaA *gene [19,21]. It has also been suggested that the origin of replication has been relocated during evolution [21]. Our analysis showed that the origin of replication was not linked to the *dnaA *gene and if the origins for all *Brachyspira *genomes were set according to the skew analysis; a distinct non-permutated X-pattern could be seen in the genome alignments (Figure 2). These new positionings of the origin of replications are also supported by the Ori-Finder program [25]. The relative positions of the *dnaA *gene and the skew-analysis based origin-of-replication are shown in Figure 3. The strand compositional asymmetry (GC- and AT-skew) is usually considered to be associated with cytocine deaminations (C->T changes) in LES as a consequence of different exposure as single-stranded DNA. However, the asymmetry can have many causes, such as transcription-coupled cytocine deaminations or repairs, differences in gene density between the strands, and residue and codon bias [26]. An analysis of the local GC-skew pattern in the *B. intermedia *genome gave oscillating values that strongly correlated with gene direction. In order to better understand the sources of the asymmetry in this genome we broke down the skew patterns into LES, LAS, and coding and noncoding regions (Table 2). The overall LES skew was shown to be strongly influenced by codon frequencies on LES and LAS. In fact, the pattern seen in a sliding window GC-skew plot was almost entirely a consequence of enrichment in the number of genes on LES, *i.e.*, a total of 1639 genes (57%) on LES compared to 1231 genes (43%) on LAS. This skew did not seem to be an effect of transcription-induced mutations but rather constraints on codons. Codons starting with G were 3.3 times more frequent than codons starting with C. Since there were more codons on LES, the result was a GC-skew. Codons starting with A were 1.8 times more frequent than codons starting with T so one would expect A enrichment on LES. However, since this was an AT-rich genome, the impact of the mutationally induced skew was stronger for AT than for GC. This counteracted the expected an enrichment, resulting in a very weak T enrichment. Intergenic regions seemed to give a more unbiased measure of mutational skew with G and T enrichment on the leading strand (Table 2). In addition, for any given codon position, there were relatively more Gs and Ts in LES than in LAS.

**Table 2:** GC- and AT-Skew for the chromosome of *B. intermedia *PWS/A^T^

Direction of replication (LES )							
		**A**	**T**	**G**	**C**	**GC-skew**^**a**^	**AT-skew**^**b**^
**GENOME**	Total all positions	1195416	1209853	478529	420990	0.06397	0.006
**NON-CODING**	Total all positions	184194	192313	51170	45319	0.06064	0.02156

Direction of transcription (Genes)							
**LES**		**A**	**T**	**G**	**C**	**GC-skew**^**a**^	**AT-skew**^**b**^
	Total all positions	632324	533506	287359	180355	0.22878	-0.08476
	Position 1	210835	119494	165285	48912	0.5433	-0.27652
	Position 2	208733	172083	67699	96001	-0.17289	-0.09624
	Position 3	212756	241929	54375	35442	0.2108	0.06416
	Synonymous position 3	61155	95518	6848	9425	-0.15836	0.21933
**LAS**		**A**	**T**	**G**	**C**	**GC-skew**^**a**^	**AT-skew**^**b**^
	Total all positions	484034	378898	195316	140000	0.16497	-0.12184
	Position 1	159165	87186	116357	36711	0.52033	-0.29218
	Position 2	153962	126972	47711	70771	-0.19463	-0.09607
	Position 3	170907	164740	31248	32518	-0.01992	-0.01837
	Synonymous position 3	49883	63604	4059	7941	-0.3235	0.1209
**LES+LAS**							
		**A**	**T**	**G**	**C**	**GC-skew**^**a**^	**AT-skew**^**b**^
	Total all positions	1116358	912404	482675	320355	0.20213	-0.10053
	Position 1	370000	206680	281642	85623	0.53373	-0.28321
	Position 2	362695	299055	115410	166772	-0.18202	-0.09617
	Position 3	383663	406669	85623	67960	0.11501	0.02911
	Synonymous position 3	111038	159122	10907	17366	-0.22845	0.17798

^a ^Calculated as (G-C)/(G+C)

^b ^Calculated as (T-A)/(T+A)

**Figure 4:**
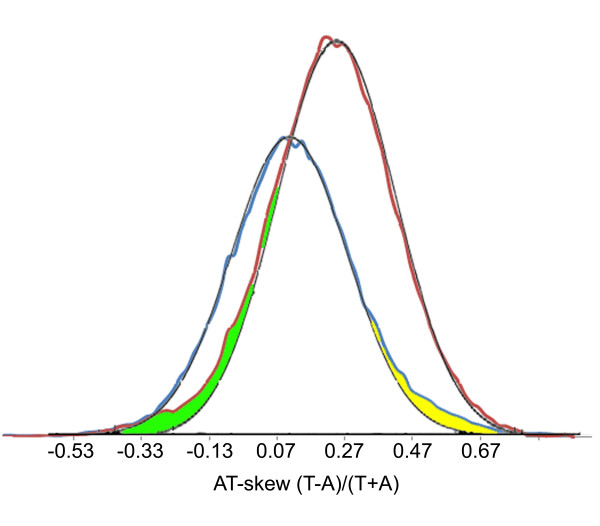
**Distribution of third codon position AT-skew of synonymous codons in leading and lagging strand genes**. AT-skew in the third position of synonymous codons used to differentiate leading (red) and lagging strand (blue) genes. Larger deviations from normal distributions are highlighted with green and yellow.

Genes can relocate symmetrically around the origin of replication as a consequence of replication-dependent rearrangements [27]. The effects of such rearrangements could be seen as an X-pattern in our alignments (Figure 2). Interestingly, genes tended to switch between LES to LAS at a certain frequency during this process, as represented by the intermixed red and blue dots in Figure 2. LES and LAS genes could partially be distinguished from each other because they had different AT-skews at the third positions of synonymous codons (Table 2). An examination of the AT-skew values of individual genes showed that they were normally distributed around the average for LES and LAS (Figure 4). Interestingly, deviations from the normal distribution were found at low skew values for LES and high skew values for LAS (Figure 4). This suggests that some high AT-skew genes had recently moved from LES to LAS and some low AT-skew genes from LAS to LES. Thus, there seemed to be equilibrium between LAS and LES gene movements that slightly favored LES. This is probably related to reduced fitness of the bacteria if certain highly expressed genes are moved to LAS because of less efficient transcription.

### The 3.2 Kb plasmid in *B. Intermedia *PWS/A^T ^is also present in *B. murdochii *56-150^T^

A 3,260 bp plasmid was found with three putative genes: Bint_4001 (48.7 KDa), Bint_4002 (15.3 kDa) and Bint_4003 (4.4 kDa). We could not assign functional annotations to these genes, but a low level of similarity was found between Bint_4002 and a *Roseburia *replication initiator protein (48% similarity over 69% of the sequence, GenBank accession no. ZP_04745519). By comparing the coverage of the plasmid and the chromosome in the assembly, we could estimate the copy number to be approximately 36. We used four PCR reactions to amplify the genes and an intergenic region to screen for the plasmid in a panel of 10 *Brachyspira *strains (Table 3). We could detect the plasmid with all four PCR reactions in *B. Intermedia* PWS/A^T^ and *B. murdochii *56-150^T^. None of the other tested strains/isolates gave PCR products. The *B. murdochii *56-150^T ^genome has been sequenced and deposited in GenBank but the plasmid has not been described [20]. We downloaded the *B. murdochii *454 reads from NCBI sequence read archive (SRA) and mapped them onto our plasmid using the Roche GS mapper. The recreated draft *B. murdochii *plasmid sequence was approximately 96% identical at nucleotide level to the *B. intermedia *plasmid. Thus, the plasmid seems not to be restricted to *B. intermedia *PWS/A^T ^but it is far from ubiquitous in *Brachyspira *isolates.

**Table 3:** PCR targeting the 3.2 kb plasmid *of Brachyspira intermedia*

***Brachyspira *sp**.	Host animal	Country	PCR
*B. hyodysenteriae *AN1409:2/01	Mallard duck	Sweden	-
*B. intermedia *PWS/A^T^	Pig	United Kingdom	+
*B. intermedia *AN2004/1/01	Chicken	Sweden	-
"*B. suanatina*" AN4859/03R	Pig	Sweden	-
*B. innocens *B256*T*	Pig	USA	-
*B. innocens *AN64/1/04	Chicken	Sweden	-
*B. murdochii *56-150^T^	Pig	Canada	+
*B. murdochii *AN1780/3/03	Chicken	Sweden	-
*B. alvinipulli *AN1268/3/04	Chicken	Sweden	-
*B. pilosicoli *P43/6/78^T^	Pig	United Kingdom	-

The 55.9 kb *B. hyodysenteriae *WA1 plasmid previously described was not present in *B. intermedia *PWS/A^T^. However, some of the *B. hyodysenteriae *WA1 plasmid genes were homologous to genes in the *B. intermedia*, *B. murdochii *and/or *B. pilosicoli *chromosomes. Several of the homologous genes showed high conservation and only 10 genes, many of which encode glycosyltransferases (Additional file 1, "plasmids" tab), were genuinely unique to the plasmid. A cluster of four genes (*rfbA, rfbB, rfbC *and *rfbD*) has been discussed previously in terms of virulence [19,22]. They were all conserved in the *B. intermedia *chromosome although one gene (*rfbA*) contained two nonsense mutations. The *rfbaA-D *genes are involved in the rhamnose biosynthesis pathway and are believed to modify the O-antigen backbone of the cell wall LOS. These data suggest that rhamnose biosynthesis capacity may be present in *B. intermedia *strains, independent of their 32 kb plasmid. However, the inactivation of the *rfbA *gene in this strain suggests it may not be essential for virulence in *B. intermedia *PWS/A^T^. It will be interesting to see if the *rfbA-D *gene cluster is present and if it is functional in other *B. intermedia strains *and correlates to pathogenicity.

### Genome comparison

**Figure 5:**
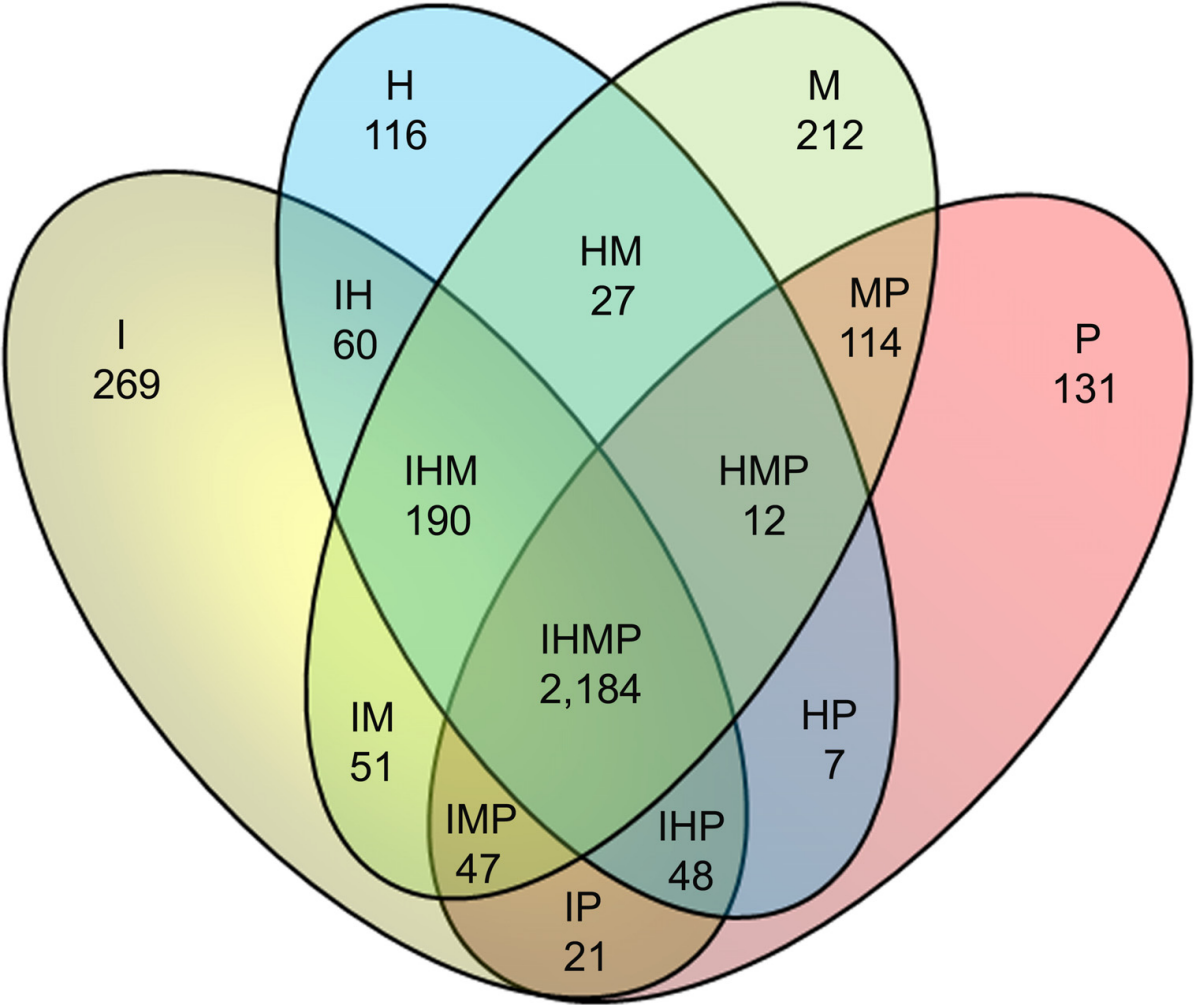
**Genome content comparison**. Venn diagram of genome content unique to and shared between *B. intermedia *(I), *B. hyodysenteriae *(H), *B. murdochii *(M) and *B. pilosicoli *(P) based on a BLASTP comparison analysis with an e-value cutoff set to 1e-9. 15 classes can be recognized (IHMP, IHM, IHP, IMP, HMP, IH, IM, IP, HM, HP, MP, I, H, M and P).

Complete genome sequences from *B. hyodysenteriae *[19], *B. pilosicoli *[21] and *B. murdochii *[20] are available and a comparative analysis between the completed *B. hyodysenteriae *and *B. pilosicoli*, and the draft version of *B. murdochii *have been made [21]. We can now add a fourth completed genome, *B. intermedia*, and also use a completed version of the *B. murdochii *genome to improve the comparisons. Thus, we have categorized the pan-genome of the *brachyspira *species *B. intermedia *(I), *B. hyodysenteriae *(H), *B. murdochii *(M) and *B. pilosicoli *(P) into 15 classes (IHMP, IHM, IHP, IMP, HMP, IH, IM, IP, HM, HP, MP, I, H, M and P) on the basis of a BLASTP e-value with a > 1e-9 cutoff. The number of genes in each class is shown in Table 4 and Figure 5. Some genes were close to the threshold cutoff value and we therefore included the e-values in the table to describe the genes so that a manual estimation can be made for individual genes (Additional file 1). In the previous comparisons 1,589 genes unique to *B. murdochii*, 703 unique to *B. hyodysenteriae *and 525 unique to *B. pilosicoli *were defined [21]. Here we make a more refined analysis of unique genetic material and can therefore reduce these numbers 4-7 fold (Table 4) and add the *B. intermedia *specific gene category and combinatory classes (Figure 5). The genomic locations of the genes from the 15 classes are shown in Figure 3. Unique genes were widespread around the genome but some clustering existed.

**Table 4:** Conserved and shared genes between the four *Brachyspira *species

	Shared	Hypotethical	Phage	Virulence	Other
**H**	116	97	1	4	14
**P**	131	95	1	3	32
**M**	212	156	0	1	55
**I**	269	207	22	3	37
**HP**	7	5	0	0	2
**IP**	21	7	2	1	11
**HM**	27	14	0	2	11
**IM**	51	36	0	0	15
**IH**	60	29	1	6	24
**MP**	114	54	23	0	37
**HMP**	12	4	0	2	6
**IMP**	47	30	0	0	17
**IHP**	48	19	1	2	26
**IHM**	190	110	1	2	77
**IHMP**	2,184	578	8	279	1,319

### Distribution of Clusters of Orthologous Genes (COG) categories

COG classifications were assigned to each protein by comparison to the COG database [28]. COG classifications have previously been made on *Brachyspira *genomes [19-21]. Our extended analysis showed that all four available *Brachyspira *genomes had a similar overall COG profile (Additional file 1, "COG1" tab). The COG assignments were also incorporated into the descriptions of the 15 conservation classes in Additional file 1, (tab 1-15) and the classes were compared in Additional file 1, "COG2" tab. The unique gene classes (I, H, M and P) were generally similar in the COGs suggesting that the main difference lies among the more poorly characterized functions. Some exceptions can be noted. The *B. murdochii *specific genes were 5-10 times more abundant in the COG "Amino acid transport and metabolism" and 2-5 times more abundant in "Replication, recombination and repair" compared to the other unique classes. The IHM class (genes lost by *B. pilosicoli*) was particularly abundant in COGs representing transport and metabolism of inorganic ions, amino acids and carbohydrates. The lower number of inorganic ion transport and metabolism genes in *B. pilosicoli *has previously been observed [21].

### Species-unique genes

**Figure 6:**
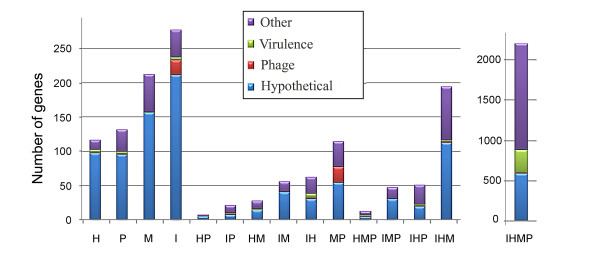
**Categorized pan genome**. The pan genome of the four Brachyspira species *B. intermedia *(I), *B. hyodysenteriae *(H), *B. murdochii *(M) and *B. pilosicoli *(P) were divided into 15 classes (IHMP, IHM, IHP, IMP, HMP, IH, IM, IP, HM, HP, MP, I, H, M and P) on the basis of a BLASTP comparison and an e-value > 1e-9 cutoff. Gene classifications are color coded as follows: red for putative phage genes, green for putative virulence genes, blue for genes coding for hypothetical proteins. The remaining genes are colored purple.

*B. intermedia *had the greatest number of unique genes followed by *B. murdochii *whereas *B. hyodysenteriae *and *B. pilosicoli *had lower numbers (Figure 6). The number of unique genes at a cutoff set to 1e-9 and 1e-4 respectively was I = 269/226, H = 116/89, M = 212/167 and P = 131/98 ("twilight" genes in the interval 1e-4 to 1e-9 are shaded grey in Additional file 1). The large number of unique *B. intermedia *genes was partly a consequence of two prophage regions. The number of *B. intermedia *specific phage annotated genes was 22, but probably 60 of the genes belong to the phages.

More specialized species usually undergo reductive evolution, thereby losing unnecessary genes [29]. The smaller number of specific genes in the more niched pathogens *B. hyodysenteriae *(swine dysenteriae) and *B. pilosicoli *(intestinal spirochaetosis) suggests they have a higher degree of specialization. Thus, the major reason for the greater number of unique genes in *B. intermedia *and *B. murdochii *is probably related to the presence of larger pan-genomes due to higher diversity within these species.

A majority of the unique genes, in all species, were annotated as coding for hypothetical proteins. This shows that, in general, the specific traits and aspects of virulence mechanisms are poorly studied. In the high-cutoff comparison (BLASTP e-value < 1e-4), only 13 functionally annotated chromosomal genes specific for *B. hyodysenteriae *were found. There were three citrate lyases, two pepdidases, an acetyltransferase, a transmembrane protein, an ethanolamine utilization protein, an Appr-1-p processing enzyme, a small MutS-related domain protein, an ankyrin repeat-containing protein, an YcfA-like protein, and an OrfC protein. The *B. hyodysenteriae *plasmid also contained several specific glycosyltransferases and hydrolases. *B. intermedia *specific genes, apart from phage-related genes, included five microcompartmental proteins, a beta-galactosidase, three glucose-1-phosphate thymidylyltransferase, several restriction enzyme system-related genes and an extracellular solute-binding protein. *B. pilosicoli *specific genes included several transporters, a peptidase, two sialidase (neuraminidase) family-like proteins, a class D beta-lactamase and a protein possibly involved in chromosome segregation. *B. murdochii *specific genes included a CRISPR system, two capsular polysaccharide biosynthesis proteins, two SNARE associated Golgi -related proteins, two STAM (AMSH)-SH3 domain associated-proteins, several transcription regulators, and DNA replication proteins.

### Genes shared by two species

One could expect that the two more specialized pathogens, *B. hyodysenteriae *and *B. pilosicoli*, would share some virulence-associated genes, but they had the least number of shared genes. Actually, only two hypothetical proteins were shared at the higher stringency level. This most likely reflects the fact that these two species seem to exploit distinctly different life strategies. The greatest number of shared genes for any pair of species was that for *B. murdochii *and *B. pilosicoli*. The high number was partly a result of a common phage found in one copy in *B. pilosicoli *and in three copies in *B. murdochii*. The phages are described in more detail below.

### Genes shared by three species (conserved genes lost by one species)

The greatest number of shared genes between three species was in the class *B. intermedia, B. hyodysenteriae *and *B. murdochii*. One could see these genes as conserved genes lost by *B. pilosicoli *during reductive evolution. In particular, *B. pilosicoli *has lost many transport-related proteins which could reflect its adaptation to a more specialized ecological niche. The higher level of reductive evolution in *B. pilosicoli *suggests it is an older pathogen than *B. hyodysenteriae*. The pathogenicity of the younger *B. hyodysenteriae *could be related to its acquisition of the 32 kb plasmid. The least number of genes was found in the *B. hyodysenteriae*, *B. murdochii *and *B. pilosicoli *category. This corresponds to conserved genes lost by *B. intermedia*. This, once again, shows *B. intermedia *to be the species with the largest number of accessory genes.

### Bacteriophages and their role in horizontal gene transfer (HGT)

A phage-like gene transfer element named VSH-1 is known to have the capacity to transduct randomly packaged genomic material between *B. hyodysenteriae *strains [23]. The *B. intermedia *VSH-1 region had a small cluster of putative VSH-1 like genes located approximately 16 kb upstream of the main part of VSH-1, similarly as previously described in *B. hyodysenteria *[30]. Here we report two new *B. intermedia *bacteriophages, pI1 and pI2 (Figure 3). pI1 was located almost completely adjacent to the VSH-1 phage-like element (Bint_0105- Bint_0143). It had a size of ~28 kb and contained 37 genes (Bint_0068- Bint_0103) of which 36 were unique to *B. intermedia *also at the high stringency level (e-value < 1e-4). Interestingly, one gene coding for a hypothetical membrane-spanning protein, Bint_0072, was conserved in *B. hyodysenteriae *(BHWA1_02012, e-value = 5e-18). This suggests that an HGT event has taken place and that the pI1 phage has acquired this gene from another *B. intermedia *or *B. hyodysenteriae *strain and transferred it to this strain.

The second phage, pI2, was ~16 kb and contained 24 genes (Bint_1512- Bint_1535) divided into three regions. The first region (Bint_1512-Bint_1525) and the third (Bint_1531- Bint_1535) were unique for *B. intermedia *also at the high stringency level. However, the second region (Bint_1526-Bint_1530) had similarities to both the VSH-1 phage-like element of the *Brachyspira *species and to the shared phage found in *B. pilosicoli *and *B. murdochii*. Thus, this phage seems to be a hybrid that has acquired new properties from other phages or VSH-1 phage-like elements. The genes in this region were annotated as hypothetical proteins and endolysin glycoside hydrolase.

**Figure 7:**
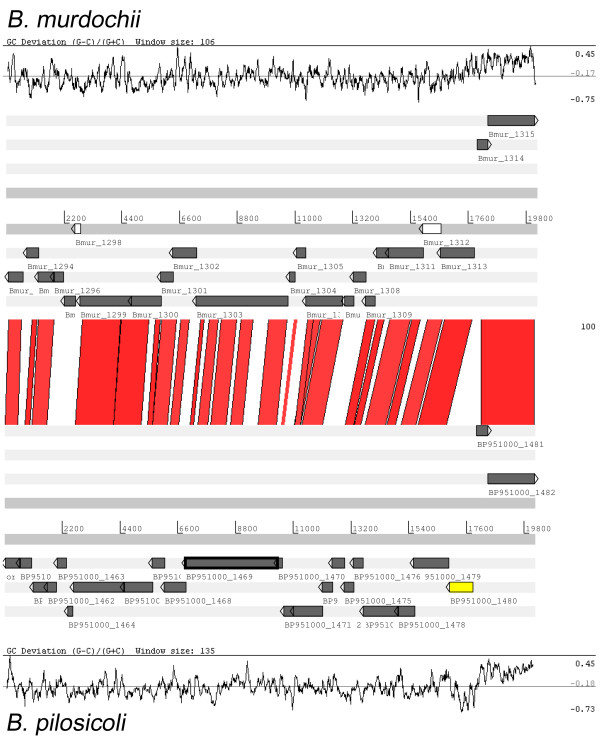
**Pairwise ACT comparison of the two phages pP1 and pM1**. Sequences are aligned with BLASTN from the predicted start of the phages and visualized in ACT at a cutoff set to 100. Pseudogenes are colored white and a gene-insertion event involving a DNA methylase gene (BP951000_1480) is yellow.

In the gene classification described above, one bacteriophage was found conserved in *B. pilosicoli *and *B. murdochii*. One copy was present in *B. pilosicoli *(pP1: BP951000_1459- BP951000_1482) and three in *B. murdochii *(pM1: Bmur_1293- Bmur_1315, pM2: Bmur_1677- Bmur_1699, pM3: Bmur_2253- Bmur_2275). Two of the *B. murdochii *phages, pM1 and pM2, were almost identical to each other while the pM3 phage showed higher variability, especially in one region. A comparison of the pP1 phage with the pM1-3 phages revealed an extra gene, BP951000_1480, that codes for a DNA methylase (Figure 7). This gene had a deviating GC-skew value compared to all other phage genes suggesting that it recently had been inserted into the phage. The DNA methylase was most related to a *Lactobacillus *DNA-methylase (GeneBank accession no.: ADF83450, e-value: 4e-51). Thus, the gene has probably been acquired from outside the *Brachyspira *genus.

It has been shown that *Brachyspira *spp. and *Clostridium *share an unexpected amount of significant gene similarity [19]. When comparing the pP1 and pM1-3 phages to sequences in GenBank, we found homology to a *Clostridium *phage in the complete genome of the *C. botulinum *strain A2 Kyoto (Additional file 2). This suggests the phages may occasionally transfer between species and genera and exchange genetic material resulting in cross-species and cross-genus HGT events.

In our analysis, we have seen several indications of putative HGT events mediated by bacteriophages. However, we still have only a single genome sequence for each species and this is a major limitation when analyzing HGT events. The rapid development of sequencing technology will hopefully give us opportunity to characterize a larger set of genomic data from *Brachyspira *spp. in the future. This will permit a more robust HGT analysis. Also the gene classification will become more robust as the dataset will grow. The accessory gene-pool is likely to increase, but the truly unique genes for each spices will likely decrease, sorting out the essential species specific factors better.

## Conclusions

In this study, we classified the accessory gene pool into different classes of shared and unique genes. In general, the unique genes are poorly annotated, which shows our lack of knowledge about species-specific processes. *B. intermedia *and *B. murdochii *contain more material specific to them and probably a larger pan-genome. *B. hyodysenteriae *and *B. pilosicoli *are both more specialized pathogens that have less accessory genetic material and diversity. They have specialized independently, as seen by the little genetic material shared only between them. These two genomes were also the two smallest, indicating that reductive evolution had taken place [31]. The traces of reductive evolution involved loss of genes, especially transport proteins. This is most obvious in *B. pilosicoli *suggesting it has been influenced by reductive evolution for a longer time. The pathogenicity of *B. hyodysenteriae *could in analogy with *Yersinia pestis *[32], be a result of the acquisition of a plasmid.

The gene content of the different classes of accessory genes is under the influence of HGT. Here we outlined a mechanism for HGT between classes that involves gene rearrangements involving bacteriophages with broad tropism. Our data also suggest cross genus HGT events could have occurred via the phages. These mechanisms need further studies if we want to understand the dynamics of the complete pan-genome of the species.

Genome sequencing of more strains will probably reduce the numbers of unique and shared features even more. Although we now have a markedly more confined list of candidate genes, the large number of annotations for "hypothetical proteins" among the unique genes shows that it is of great importance to study gene functions in order to understand species-specific traits.

## Methods

### Bacterial culture, DNA preparation and 454 sequencing

The *B intermedia *type strain PWS/A^T ^was obtained from the American type culture collection (ATCC^®^51140). The strain was cultured twice on fastidious anaerobe agar plates supplemented with 10% equine blood (FAA). The purity of the strain was assessed by phase-contrast microscopy. Genomic DNA was prepared using the Qiagen DNAeasy kit. Parallel sequencing was performed using the Roche 454 FLX platform. One half picotiterplate of shotgun sequences was produced and *de novo *assembled using the GS assembler (Newbler). In total, 193,367 reads with an average length of 245 bp were assembled into 203 contigs with sizes ranging between 100 and 138,989 bp. The average coverage was 14X. Contigs were handled by the Consed package [33]. Gaps, uncertain regions, and misassemblies were closed and solved manually by PCR, Sanger sequencing, and local reassemblies. The estimated average sequence quality as reported by Consed, based on the Q values of the reads (-10log(Pe)) was 1.5 errors in 10,000 bp. Sequence reactions were performed with the BigDye^® ^Terminator v.3.1 kit (Applied Biosystems) and analysed in an ABI PRISM^® ^3100 Genetic Analyzer at Applied Biosystems, Carlsbad, CA, USA. A total of 218 Sanger reads were incorporated into the assembly.

### Sequence analysis and annotation

Genes were predicted using Glimmer 3 [34]. Annotations were handled by the Artemis software [35]. Conserved genes for *B. intermedia, B. hyodysenteriae, B. murdochii and B. pilosicoli *were automatically annotated by comparison at protein level and transfer of annotation data. Less conserved genes were queried against the proteins of all microbial genomes and NCBI nr. Manual inspections were made when similarities were weak. tRNAscan-SE [36] was used to identify tRNAs genes and rRNA genes were defined by their similarity to other *Brachyspira *sequences. Circular DNA plots of the *B. intermedia *genome and plasmid were drawn with DNAplotter [37]. Genome alignments were made with Mummer [38] and ACT [39].

Complete nucleotide sequences and annotations of the *B. intermedia *chromosome and plasmid have been deposited in the GenBank database, accession numbers CP002874 (chromosome) and CP002875 (plasmid).

The skew analysis was made by functions in Artemis and DNAplotter and by in-house made scripts. LES and LAS genes were defined and exported in Artemis and a perl script was used to count the number of a/c/t and g's in each codon position. The whole genome was also analyzed using a perl script where LES and LAS were separated based on the positioning of *oriC *and the genome feature file was used to identify non-coding regions.

### Unique and shared genes

All-against-all protein sequence comparisons were made and genome-specific best hits (BeTs) were calculated [28]. A BeT was considered to represent conservation between two proteins if the e-value was lower than 1e-9. The BeTs were used to assign each gene to a class using a perl script that compared all BeTs from every protein to all proteomes in the analysis. The results were collected in Additional file 1. Genes were compared using BLASTP. Genes with a best hit e-value < 10-9 were considered to be conserved. Gene pairs with e-values between 1e-4 and 1e-9 were considered to be in a "twilight zone". Genes were classified into 15 classes (IHMP, IHM, IHP, IMP, HMP, IH, IM, IP, HM, HP, MP, I, H, M and P) on the basis of the BLASTP values. Functional classification into phage and putative virulence factors were made by manual inspection.

### Plasmid analysis

The plasmid was purified with a standard plasmid miniprep protocol. The PCR amplifications of plasmid genes were made with the following primer-pairs G1*f *5'-CAATTTTAATGCTAAGACTTTGAA-3', G1*r *5'-CGCTTTAATGTTCTATTCGG-3', G2*f *5'-GTTTTACCTTTCATATCATCACAA-3', G2*r *5'-TTTTCTGTCGTCATTATCTTTTC-3', G3*f *5'-GACTAACGCACCGACAATAAT-3', G3*r *5'-AATTCTTAATAGTTGCCTTTCAGTA-3'. The following templates were used: *B. intermedia *PWS/A^T ^*, B. intermedia *AN2004/1/01, *B. hyodysenteriae* AN1409:2/01*, B. "suanatina" *AN4859/03^R ^*, B. innocens *B256^T^, *B. innocens *AN64/1/04, *B. murdochii *56-150^T ^*B. murdochii *AN1780/3/03, *B. alvinipulli *AN1268/3/04 and *B. pilosicoli *P43/6/78^T^.

### Genomic comparisons

Average Similarity of the conserved Core method (ASC) [24] was used to measure the phylogenomic distance between the complete genomes of *B. intermedia *PWS/A^T^*, B. hyodysenteriae *WA1, *B. murdochii *56-150^T^, *B. pilosicoli *95/1000 and *Leptospira interrogans *(AE016823-24). A dendrogram was created by converting a similarity matrix to a distance matrix and calculating a tree rooted using *Leptospira interrogans as outgroup species *with the UPGMA method using PHYLIP 3.67 through the Mobyle platform (http://mobyle.pasteur.fr/). A phylogenetic tree was then plotted using PhyloDraw (http://www.bioinformatics.org/wiki/PhyloDraw).

### Distribution of Clusters of Orthologous Genes (COG) categories

All proteins from all four *Brachyspira g*enomes in the analysis were compared with the COG database [28]. BeTs were identified and COG classes were assigned to all proteins matching the COG database with an e-value below 1e-9. The COGs for each species and gene content class were summarized in a table (Additional file 1) and inspected to identify conservation patterns of specific functions.

## Competing interests

The authors declare that they have no competing interests.

## Authors' contributions

BS and TH conceived and designed the study. TH performed the gap closure and annotated the genome. BS and TH analyzed the skew. TH performed comparative analysis. TH analyzed plasmids and DJ contributed. DJ contributed with Brachyspira specific data interpretations. BS and TH wrote the manuscript and DJ contributed. All authors have read and approved the final manuscript.

## Supplementary Material

Additional file 1 **Pan genome classification**. Additional file 1 contains the 15 classes of shared and unique genes between the four Brachyspira species chromosomes: *B. intermedia *PWS/A^T^, *B. hyodysenteriae *WA1, *B. murdochii *56-150^T ^and *B. pilosicoli *95/1000 and plasmids genes comparison. Click here for file

Additional file 2 **ACT comparison of the phage pP1 and the phage in *Clostridium botulinum *str A2 Kyoto**. Sequences were aligned with TBLASTX from the predicted start and visualized with ACT at a cutoff set to score 200. Blue lines link matches in reverse orientation and red matches oriented in the same direction. Click here for file
