# Supplementary figures and images for: Complete genome sequence of Brachyspira intermedia reveals unique genomic features in Brachyspira species and phage-mediated horizontal gene transfer
Source: BMC Genomics. 2011 Aug 4;12:395. doi: 10.1186/1471-2164-12-395 (PMC3163572; doi:10.1186/1471-2164-12-395)

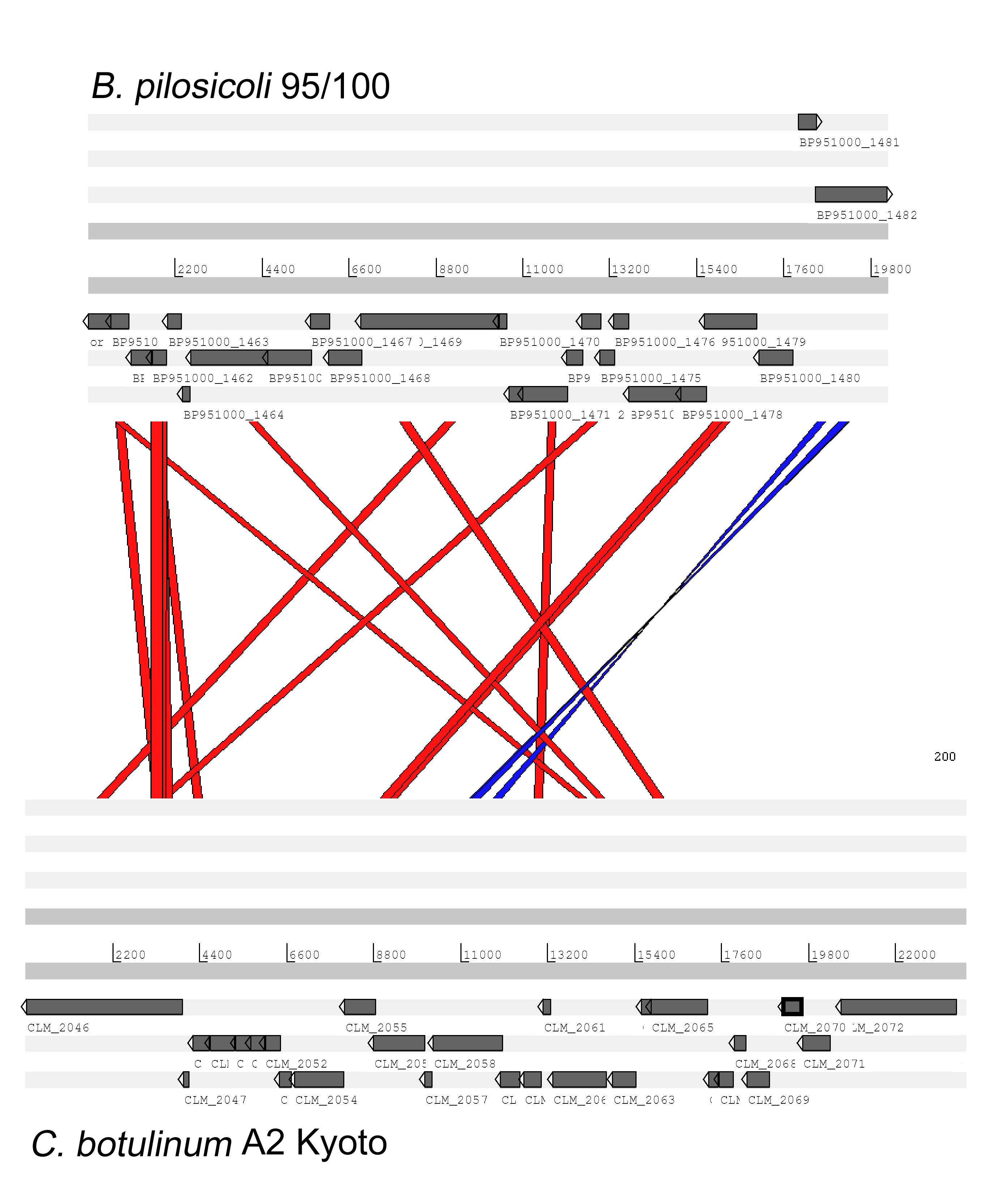

Supplement: Additional file 2 — ACT comparison of the phage pP1 and the phage in Clostridium botulinum str A2 Kyoto. Sequences were aligned with TBLASTX from the predicted start and visualized with ACT at a cutoff set to score 200. Blue lines link matches in reverse orientation and red matches oriented in the same direction. [file 1471-2164-12-395-S2.TIFF]
